# RNA-Binding Protein IGF2BP1 Associated With Prognosis and Immunotherapy Response in Lung Adenocarcinoma

**DOI:** 10.3389/fgene.2022.777399

**Published:** 2022-01-27

**Authors:** JinFeng Liu, Zhi Li, Iokfai Cheang, Jinghang Li, Chunlei Zhou

**Affiliations:** ^1^ Department of Immunology, School of Medicine and Holistic Integrative Medicine, Nanjing University of Chinese Medicine, Nanjing, China; ^2^ Department of Cardiovascular Surgery, The First Affiliated Hospital of Nanjing Medical University, Nanjing, China; ^3^ Department of Cardiology, The First Affiliated Hospital of Nanjing Medical University, Nanjing, China; ^4^ Department of Pathology, Children’s Hospital of Nanjing Medical University, Nanjing, China

**Keywords:** lung adenocarcinoma, TCGA, m^6^A modification regulators, prognosis, immunotherapy response

## Abstract

N6-methyladenosine (m^6^A) is the most common modification in eukaryotic RNAs and plays a vital role in the tumorigenesis and metastasis of various cancers. However, a comprehensive study of m^6^A methylation regulators in lung adenocarcinoma (LUAD) is still lacking. The present study aimed to systematically explore the role of m^6^A methylation regulators in LUAD. RNA sequencing data of 20 m^6^A methylation regulators and clinical data of LUAD patients were downloaded from The Cancer Genome Atlas (TCGA) database. The prognosis value of m^6^A methylation regulators in LUAD was evaluated using the Gene Expression Profiling Interactive Analysis (GEPIA) and PrognoScan database. The correlation between *IGF2BP1* and immune infiltrates in LUAD was investigated via CIBERSORT and Tumor Immune Estimation Resource (TIMER). A total of 15 m^6^A modification regulators were significantly abnormally expressed in LUAD tissues. Survival analysis revealed that four genes (*HNRNPC*, *HNRNPA2B1*, *IGF2BP1*, and *IGF2BP3*) were significantly associated with poor prognosis in LUAD. Multivariate Cox regression analysis showed that only *IGF2BP1* was an independent predictor of LUAD after adjusting common clinical parameters. The mutation rates of m^6^A modification regulators in LUAD were less than 10%. Further analysis revealed that *IGF2BP1* expression was significantly correlated with immune infiltration, the expression of immune checkpoints, and tumor mutational burden (TMB) in LUAD. Our findings suggest that *IGF2BP1* is an independent predictor and related to immunotherapy response in LUAD, which maybe a potential novel biomarker for LUAD prognosis and the status of tumor immunity.

## Introduction

Lung cancer is one of the most common malignancies worldwide. Lung adenocarcinoma (LUAD) is the main subtype of lung cancer. The development of lung cancer is the result of the combined effect of genetic and environmental factors. Despite the advancement of surgery, radiotherapy, chemotherapy, and targeted therapy, it remains a high incidence and low overall 5-year survival (Bray et al., 2018). Therefore, early diagnosis and prognostic evaluation are urgently needed to be performed in LUAD.

N6-methyladenosine (m^6^A) is the most prevalent and abundant transcriptional modification in eukaryotic RNAs and plays a key role in the process of cell self-renewal and differentiation (Desrosiers et al., 1974). The m^6^A modification is highly conservative, which is commonly found in 3’untranslated region (UTR), protein coding sequences (CDS), and transcription starting site (TSS). It regulates the posttranscriptional level of mRNA without changing the base sequence (Niu et al., 2013). The m^6^A modification is dynamically and reversibly regulated by different regulators, including m^6^A methyltransferase (“writers”), m^6^A demethylase (“erasers”), and m^6^A-binding protein (“readers”). The m^6^A-modified mRNA can be specifically recognized and bound by the m^6^A-binding protein, thereby regulating the RNA maturation, splicing, transport, degradation, and translation (Maity and Das, 2016). The abnormality of m^6^A modification can lead to the occurrence of many human diseases, such as tumors, metabolic diseases, and neurological diseases (He et al., 2019; Zhang et al., 2021).

Previous studies have shown the disorders of the m^6^A component, and the abnormal modification process can lead to the overexpression or inactivation of downstream oncogenes or tumor suppressor genes in various tumors (Zhou et al., 2020). A recent study showed that METTL3 could reduce the stability of *SOCS2* mRNA through the m6A-YTHDF2-dependent pathway. Knockdown of METTL3 could suppress cell proliferation in gastric cancer cells (Jiang et al., 2020). Additionally, downregulation of FTO could inhibit the proliferation and differentiation capacity through reducing the abundance of m^6^A in acute myeloid leukemia (AML). The inhibitors and regulators of m^6^A modification regulators have been explored as therapeutic approaches for treating cancer, such as FTO inhibitors (including rhein, R-2HG, IOX3, and FB23) and METTL3/METTL14 inhibitors (3-deazaadenosine) (Zhou et al., 2020). However, a systematical analysis of the impact of m^6^A modification regulators on LUAD is still lacking.

Our study aims to systematically analyze the expressions of m^6^A modification regulators in LUAD and explore the prognostic value and the relationships with tumor immune, which might be novel targets for the diagnosis and treatment of LUAD.

## Methods

### Datasets

The RNA-seq transcriptome data (format: HTSeq-FPKM) and corresponding clinical information of 513 LUAD samples and 59 normal samples were downloaded from The Cancer Genome Atlas (TCGA) database (https://cancergenome.nih.gov). The impact of m^6^A modification regulators on LUAD was evaluated using Gene Expression Profiling Interactive Analysis (GEPIA), PrognoScan database, cBioPorta, CIBERSORT, Tumor Immune Estimation Resource (TIMER), and gene set enrichment analysis (GSEA) databases.

### Differential Expression Analysis of m^6^A Modification Regulators

We totally selected 20 m^6^A modification regulators to analyze, including two “erasers” (*ALKBH5* and *FTO*), eleven “readers” (*HNRNPA2B1*, *HNRNPC*, *IGF2BP1*, *IGF2BP2*, *IGF2BP3*, *RBMX*, *YTHDC1*, *YTHDC2*, *YTHDF1*, *YTHDF2*, and *YTHDF3*), and seven “writers” (*METL14*, *METL3*, *RBM15*, *RBMWTAP*, *VIRMA*, *WTAP*, and *ZC3H13*). The expressions of m^6^A modification regulators in LUAD and normal lung tissues were assessed using the Wilcox test. The heatmap and scatter plot were used to display the different expressions of the 20 m^6^A methylation regulators in LUAD and normal lung tissues by R software (version: 4.0.3). *p* < 0.05 was considered statistically significant.

### Immunohistochemistry

We also analyzed the protein level of IGF2BP1 and CD20 in LUAD tissues by immunohistochemistry (IHC). A total of 30 specimens were obtained from the First Affiliated Hospital of Nanjing Medical University in China between December 2020 and January 2020, including 24 LUAD tissues and 6 normal lung tissues. The histological evaluation was performed on hematoxylin and eosin–stained sections. The LUAD tissue sections were immunostained with the primary antibody against IGF2BP1 (Proteintech, Ca#22803-1-AP, 1:100) and CD20 (Proteintech, Ca# 60271-1-Ig, 1:1,000) at 37°C. The degree of immunostaining was based on staining intensity and percentage of cells stained. The study was approved by the hospital’s Institutional Review Board. Written informed consent was obtained from all participants or their guardians before the study.

### Prognostic Value of m^6^A Modification Regulators

The Gene Expression Profiling Interactive Analysis (GEPIA) (http://gepia.cancer-pku.cn/index.html) was used to evaluate the prognosis value of m^6^A modification regulators in LUAD patients. The GEPIA database is an interactive web that includes 9,736 tumors and 8,587 normal samples from TCGA and the GTEx projects. GEPIA was used to generate survival curves, based on gene expression with the log-rank test and the Mantel–Cox test in 33 different types of cancers. The correlation between m^6^A modification regulators and survival in LUAD was further analyzed by the PrognoScan database (http://www.abren.net/PrognoScan/) based on the GEO database (GSE31210). PrognoScan searches for relationships between gene expression and patient prognosis (such as overall survival), across a large collection of publicly available cancer microarray datasets. Adjusting the prognostic variables (age, gender, smoking history, pT staging, and pN staging of the TNM classification), multivariate Cox regression analysis was used to analyze the correlation between m^6^A modification regulators and the prognosis of LUAD as well. A nomogram was used to predict the overall survival (1, 3, and 5 years) of LUAD patients. *p* < 0.05 is considered statistically significant.

### Genetic Alteration Analysis

The cBioPortal for Cancer Genomics (http://www.cbioportal.org/) is a comprehensive gene database, including different datasets such as DNA mutation, gene amplification, and methylation. Four studies from the cBioPortal database were enrolled: LUAD (Broad, Cell 2012), LUAD (OncoSG, Nat Genet 2020), LUAD (TCGA, Firehose Legacy), and Non-Small Cell Cancer (MSKCC, Cancer Discov 2017). A total of 1989 LUAD samples were used to analyze the genetic variation of m^6^A modification regulators in LUAD. Gene mutations included the following types: inframe mutation, missense mutation, splice mutation, truncating mutation, amplification, and deep deletion.

### Correlation Between *IGF2BP1* Gene and Immune Cell Infiltration

The CIBERSORT algorithm (https://cibersort.stanford.edu/) was employed for estimating the fractions of 22 phenotypes of immune cells based on gene expression profiles. In this study, the CIBERSORT database was used to explore the correlation between *IGF2BP1* and immune cell infiltration. Patients were divided into high-expression group and low-expression group according to the median value of *IGF2BP1* expression. The difference of immune cell infiltration between the two groups was evaluated by the Wilcoxon test. *p* < 0.05 is considered statistically significant.

TIMER is a comprehensive resource for systematic analysis of immune infiltrates across diverse cancer types (https://cistrome.shinyapps.io/timer/). TIMER applies a deconvolution previously published statistical method to infer the abundance of tumor-infiltrating immune cells from gene expression profiles. The TIMER database includes 10,897 samples across 32 cancer types from TCGA database. We analyzed the correlations between *IGF2BP1* expression and gene markers of tumor-infiltrating immune cells in LUAD *via* correlation modules. The gene markers of tumor-infiltrating immune cells included markers of CD4^+^ T cells, B cells, monocytes, M2 macrophages, and dendritic cells. The gene expression level was displayed with log2 RSEM.

### Correlation Between *IGF2BP1* Gene and TMB, MSI, and Immune Checkpoints

In our study, patients were divided into high-expression group and low-expression group according to the median value of *IGF2BP1* expression. Then, the differences of tumor mutational burden (TMB), microsatellite instability (MSI), and immune checkpoints between the two groups were evaluated by the Wilcox test. *p* < 0.05 is considered statistically significant.

### GSEA and Functional Enrichment of the *IGF2BP1* Gene

Gene set enrichment analysis (GSEA) by LinkedOmics (http://www.linkedomics.org/login.php) was applied to study the function of *IGF2BP1* and related signal pathways in LUAD. In addition, Gene Ontology (GO) enrichment analysis was also used to regard the possible function of the *IGF2BP1* gene in LUAD based on *IGF2BP1*-related genes.

## Results

### Differential Expression Analysis of m^6^A Modification Regulators

**FIGURE 1:**
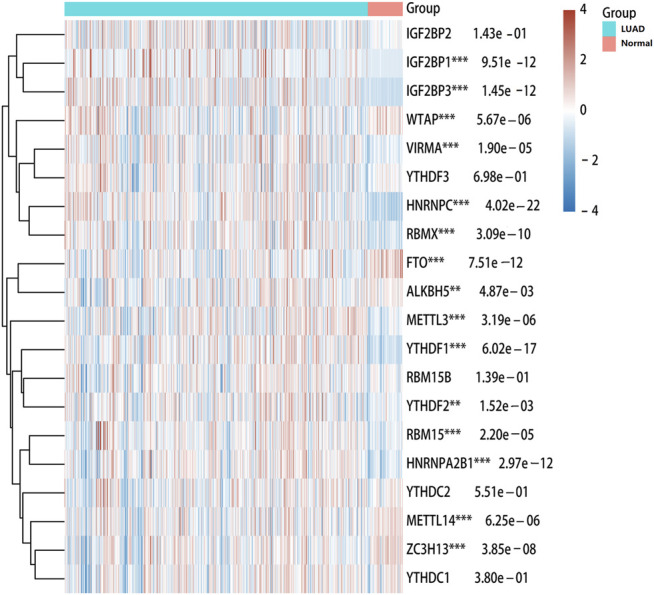
Heatmap of expressions of m^6^A modification regulators between LUAD and normal samples. The different colors represent the expression trend in different samples. ***p < 0.001, **p < 0.01, *p < 0.05.

**FIGURE 2:**
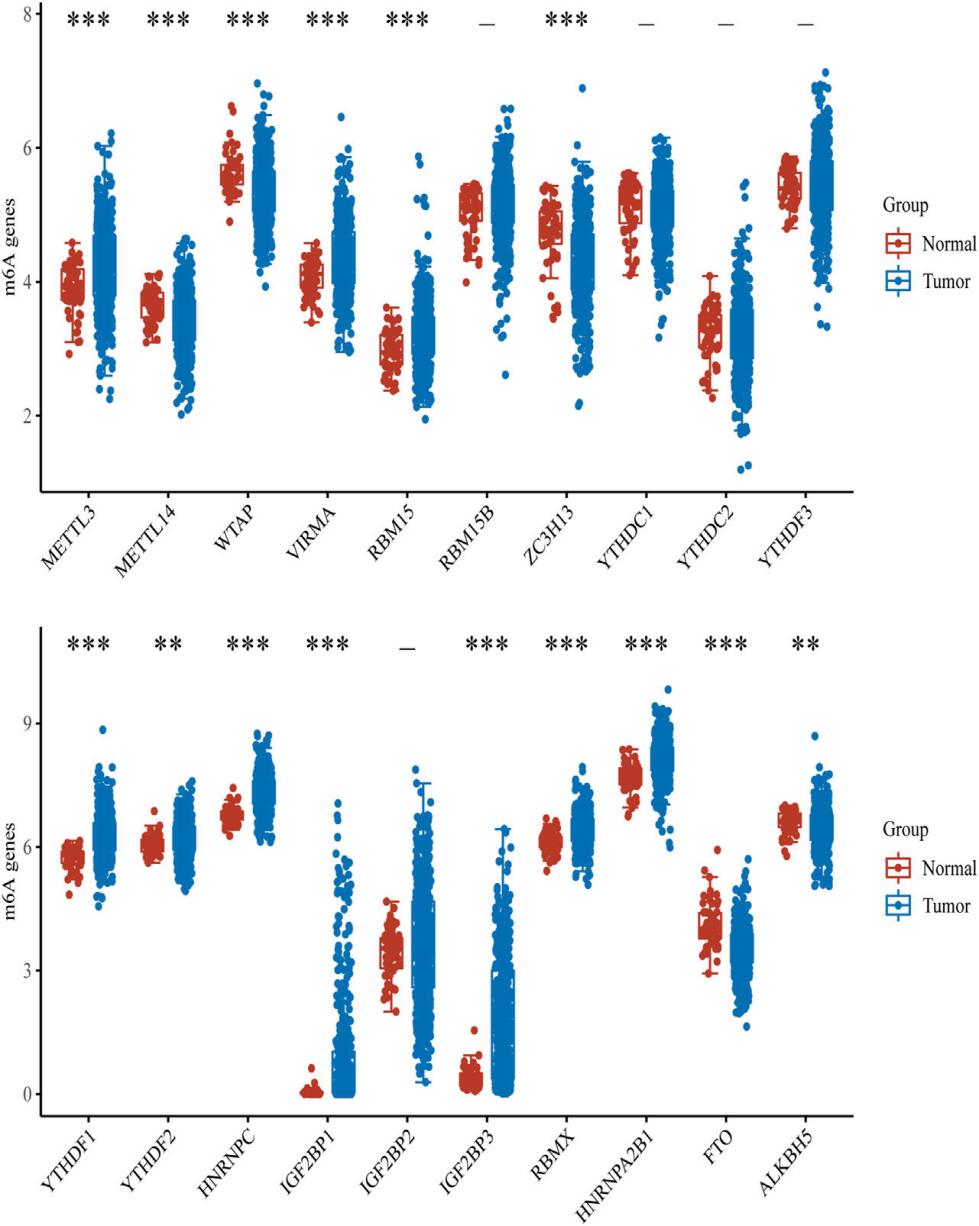
Box diagram of expressions of m^6^A modification regulators between LUAD and normal samples. Red means normal tissue, and blue means tumor tissue. ***p < 0.001, **p < 0.01, *p < 0.05.

To explore the role of m^6^A modification regulators in LUAD tumorigenesis, we systematically analyzed the expression patterns of 20 m^6^A modification regulators in LUAD tumor and normal lung tissues based on TCGA database. The heatmap for the expressions of m^6^A methylation regulators in normal and LUAD tissues showed significant differences in 15 m^6^A modification regulators (*ALKBH5*, *FTO*, *HNRNPA2B1*, *HNRNPC*, *IGF2BP1*, *IGF2BP3*, *RBMX*, *YTHDF1*, *YTHDF2*, *METL14*, *METL3*, *RBM15*, *VIRMA*, *WTAP*, and *ZC3H13*) (Figure 1). Compared with the normal tissues, the expressions of *HNRNPA2B1*, *HNRNPC*, *IGF2BP1*, *IGF2BP3*, *RBMX*, *YTHDF1*, *YTHDF2*, *METL3*, *RBM15*, and *VIRMA* were upregulated in LUAD tissues, while the expressions of *ALKBH5*, *FTO*, *METL14*, *WTAP*, and *ZC3H13* were downregulated in LUAD tissues (Figure 2). In addition, we verified the protein expression level of IGF2BP1 in LUAD tissues by IHC. The result showed that the IGF2BP1 protein expression level was significantly increased in LUAD tissues compared to that in normal lung tissues (Supplementary Figure S1), which was consistent with the mRNA level of *IGF2BP1*. These results suggested that the m^6^A methylation regulators played a vital role in LUAD.

### Prognostic Value of m^6^A Modification Regulators

**FIGURE 3:**
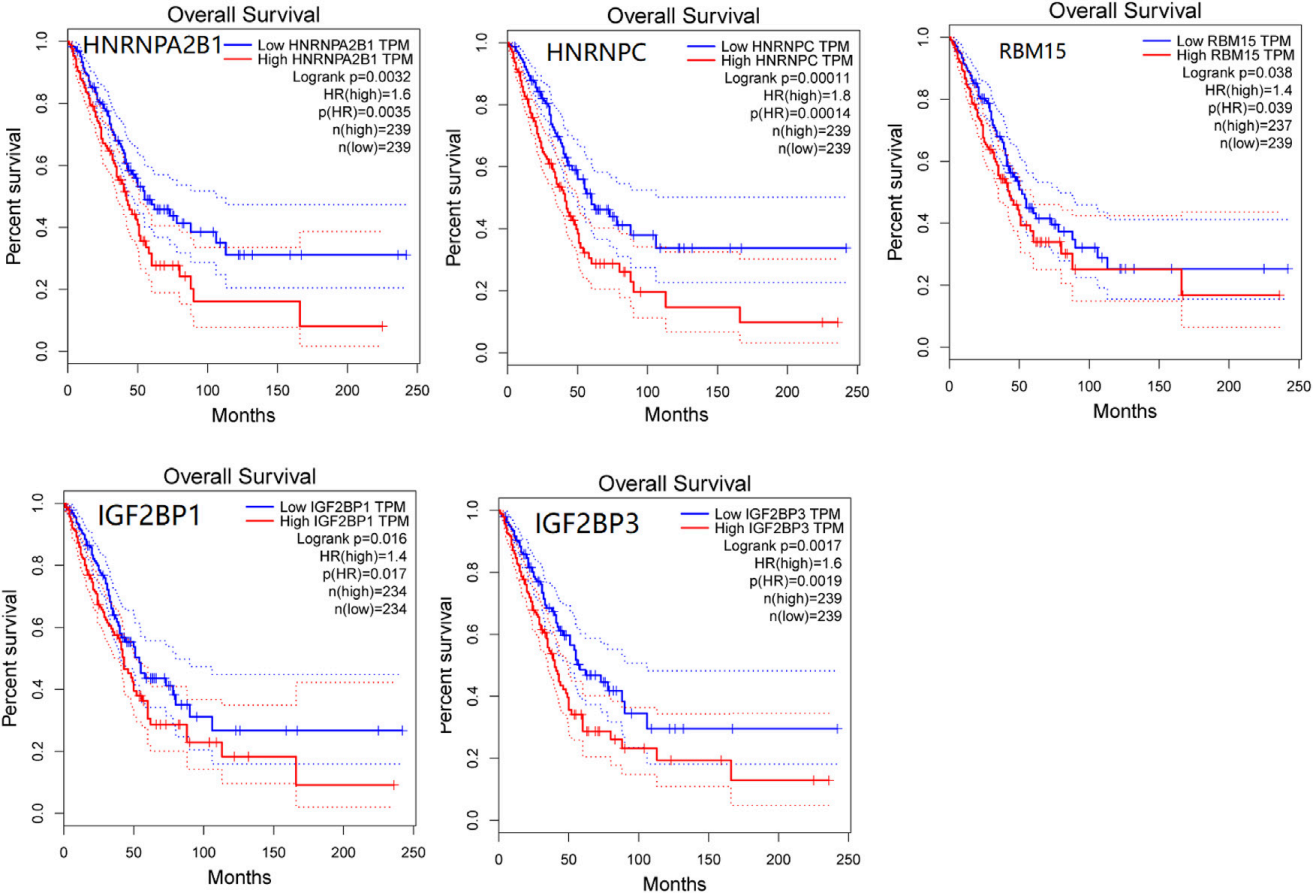
Survival analysis of m^6^A modification regulators in LUAD patients by the GEPIA database. Five genes that have significant association with poor prognosis of LUAD were presented (*p* < 0.05).

We used the GEPIA database to evaluate the prognosis value of m^6^A modification regulators in LUAD patients. The overall survival analysis revealed that the expressions of five genes were significantly associated with the poor prognosis of LUAD, including *HNRNPA2B1* (HR = 1.6, *p* = 0.0032), *HNRNPC* (HR = 1.8, *p* = 0.00011), *RBM15* (HR = 1.4, *p* = 0.038), *IGF2BP1* (HR = 1.4, *p* = 0.016), and *IGF2BP3* (HR = 1.6, *p* = 0.0017) (Figure 3). Furthermore, the PrognoScan database was also used to evaluate the prognostic value of m^6^A modification regulators in LUAD. The result showed that *HNRNPA2B1* (HR = 12.25, *p* = 0.020373), *HNRNPC* (HR = 5.77, *p* = 0.004359), *IGF2BP1* (HR = 1.59, *p* = 0.037049), and *IGF2BP3* (HR = 1.50, *p* = 0.002818) were significantly associated with the poor prognosis of LUAD (Supplementary Figure S2). These results confirmed the prognostic value of m^6^A modification regulators in LUAD.

**FIGURE 4:**
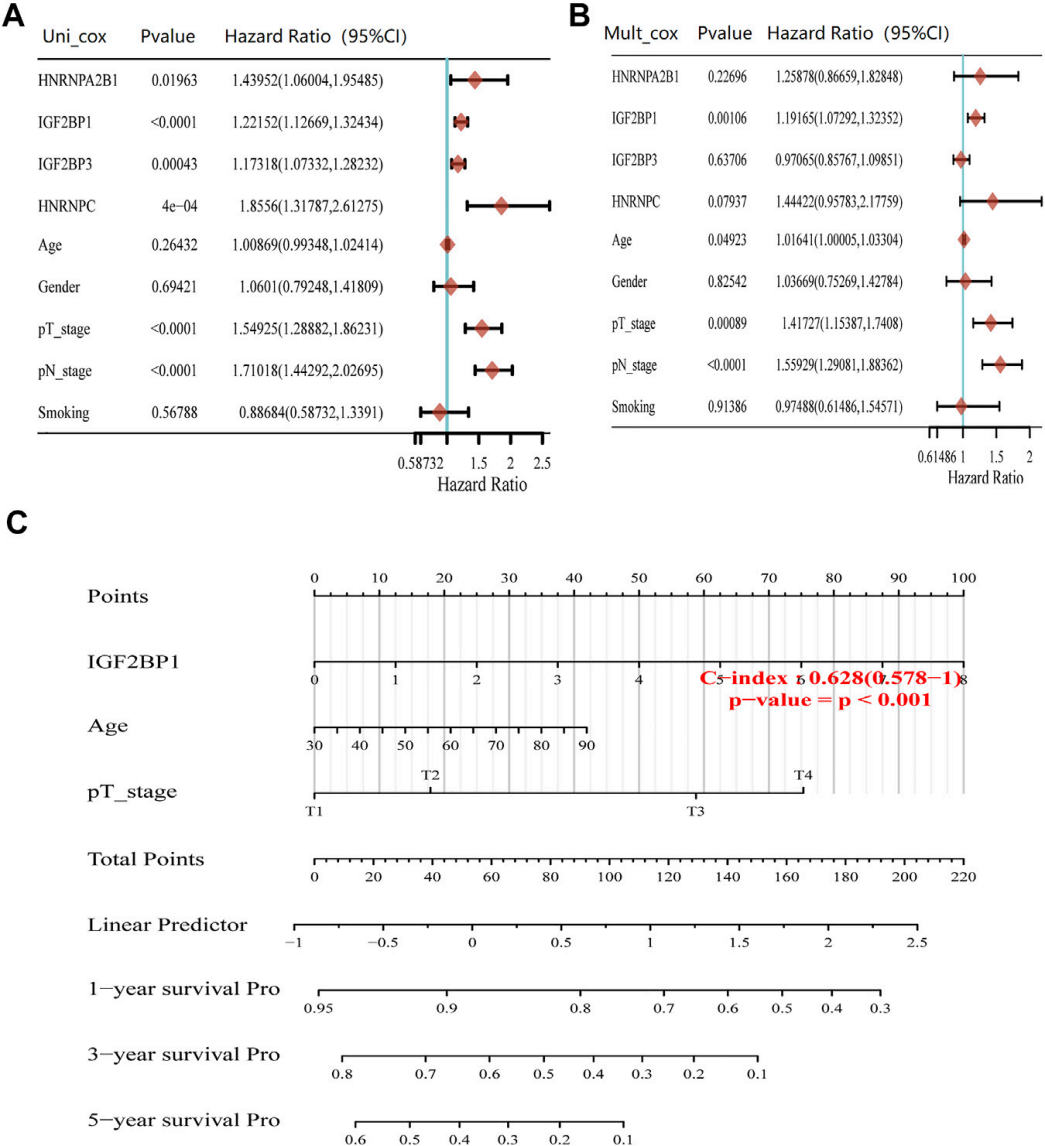
Prognosis value of m^6^A modification regulators in LUAD. **(A)** Forest plots for hazard ratios of univariate Cox regression analysis of m^6^A modification regulators and clinical relative factors in LUAD. **(B)** Forest plots for hazard ratios of multivariate Cox regression analysis of m^6^A modification regulators and clinical relative factors in LUAD. **(C)** Nomogram to predict the overall survival at 1, 3, and 5 years of m^6^A modification regulators and clinical relative factors in LUAD.

Cox regression analysis showed that *HNRNPA2B1*, *IGF2BP1*, *IGF2BP3*, *HNRNPC*, and pT/pN staging were significantly associated with the prognosis of LUAD (Figure 4A). Multivariate Cox regression analysis revealed that *IGF2BP1* (adjust HR = 1.19, 95%CI = 1.07–1.32, *p* = 0.001), pT staging (adjust HR = 1.42, 95%CI = 1.15–1.74, *p* < 0.001), and pN staging (adjust HR = 1.56, 95%CI = 1.29–1.88, *p* < 0.001) remained as the independent prognostic indicators of LUAD (Figure 4B). The overall survival analysis of LUAD patients by nomogram showed that *IGF2BP1* [C−index: 0.628 (0.578–1), *p* < 0.001] had predictive values (Figure 4C). Therefore, our finds suggested that high *IGF2BP1* expression was an independent risk factor of poor prognosis in LUAD.

### Variation of m^6^A Modification Regulators

**FIGURE 5:**
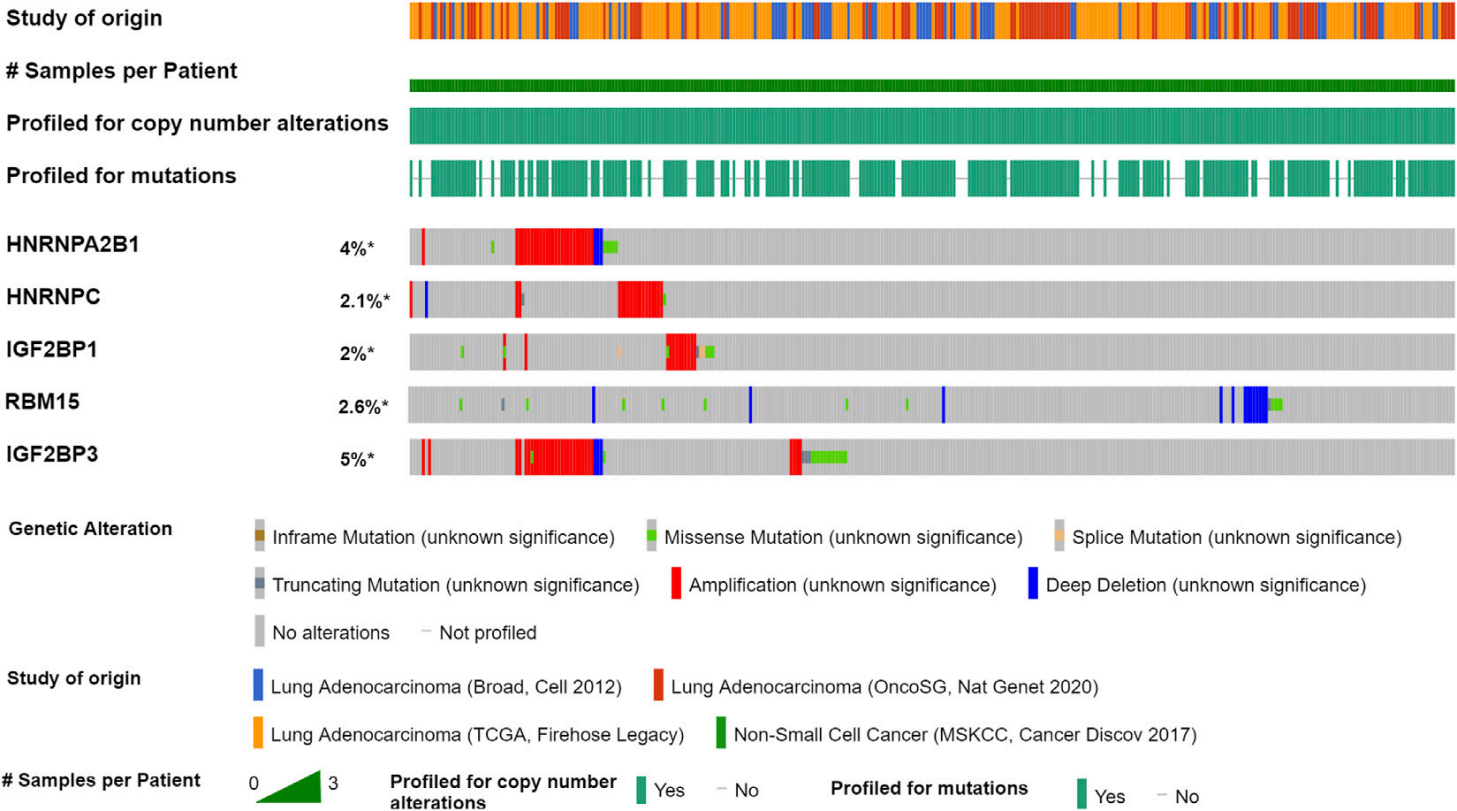
Mutation of m^6^A modification regulators in LUAD by the cBioPortal database. Four studies were enrolled: LUAD (Broad, Cell 2012), LUAD (OncoSG, Nat Genet 2020), LUAD (TCGA, Firehose Legacy), and Non-Small Cell Cancer (MSKCC, Cancer Discov 2017). A total of 1,989 LUAD samples were used to analyze the genetic variation of m^6^A modification regulators in LUAD.

We further explored the mutation rate of the significant genes (*HNRNPA2B1*, *HNRNPC*, *RBM15*, *IGF2BP1*, and *IGF2BP3*) using the cBioPortal database. The result showed that the five genes in LUAD samples had a low mutation rate (<10%) (Figure 5). Regarding the mutation type, amplification was the most predominant type for all samples. The result suggested that m^6^A modification regulators might not only influence tumorigenesis of LUAD through gene mutation.

### Correlation Between *IGF2BP1* Expression and Immune Cell Infiltration

**FIGURE 6:**
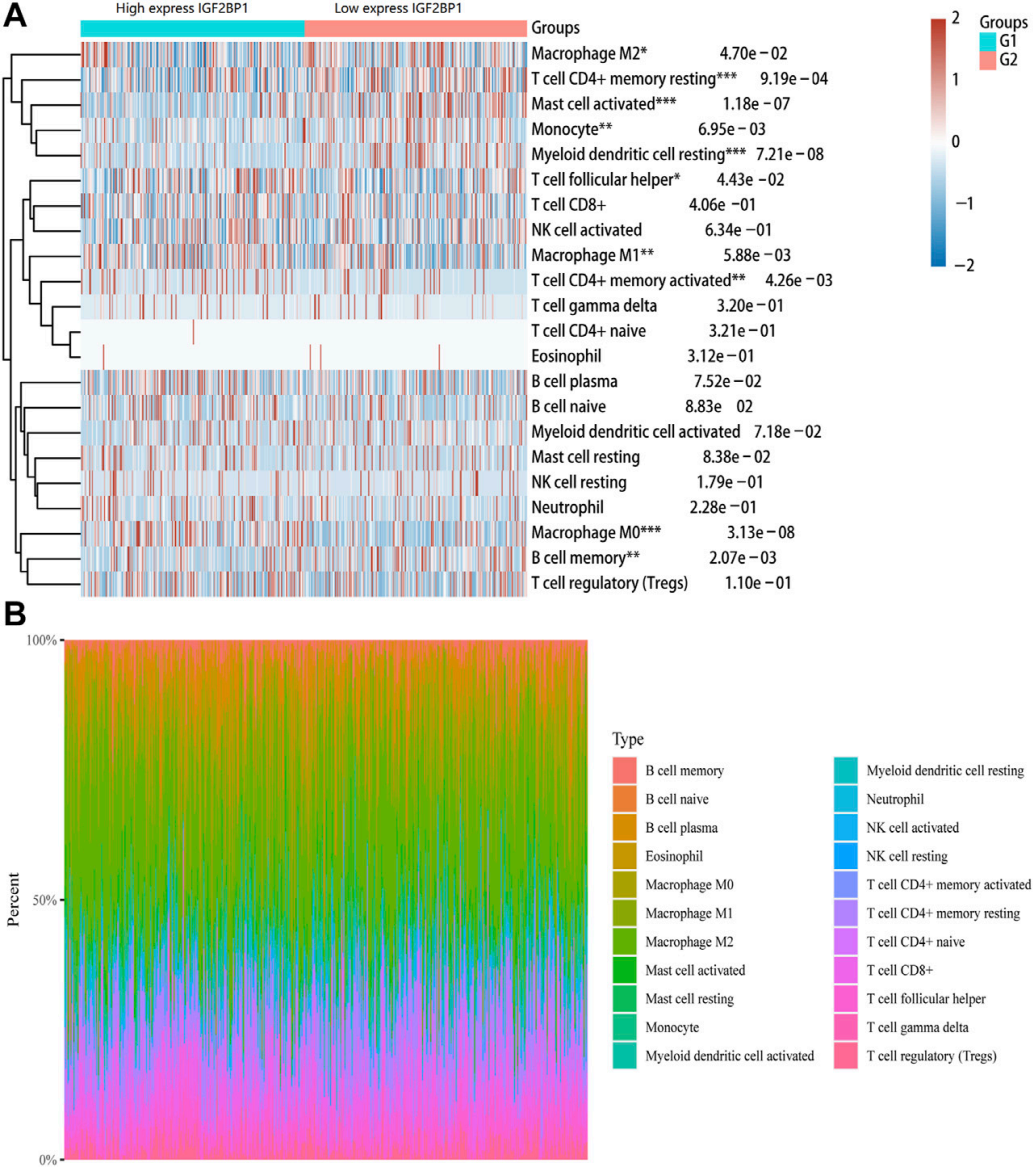
Correlation between *IGF2BP1* gene and immune cell infiltration in LUAD. **(A)** Immune cell infiltration score (CIBERSORT) heatmap, in which different colors represent the expression trend in different samples. **(B)** Percentage abundance of tumor-infiltrating immune cells in each sample, with different colors and different types of immune cells.

The tumor microenvironment is now widely considered as an important regulator of cancer progression and therapeutic response. Therefore, we investigated whether *IGF2BP1* expression was correlated with immune infiltration levels in LUAD. The immune abundances of 22 leukocyte subtypes in each LUAD sample were calculated based on CIBERSORT algorithm. The result showed that *IGF2BP1* expression was significantly associated with the infiltration of macrophage M0/1/2, T cell CD4^+^ memory resting/activation, mast cell activation, monocyte, myeloid dendritic cell resting, T cell follicular helper, and B cell memory in LUAD (Figure 6).

### Correlation Between *IGF2BP1* Expression and Immune Markers

**FIGURE 7:**
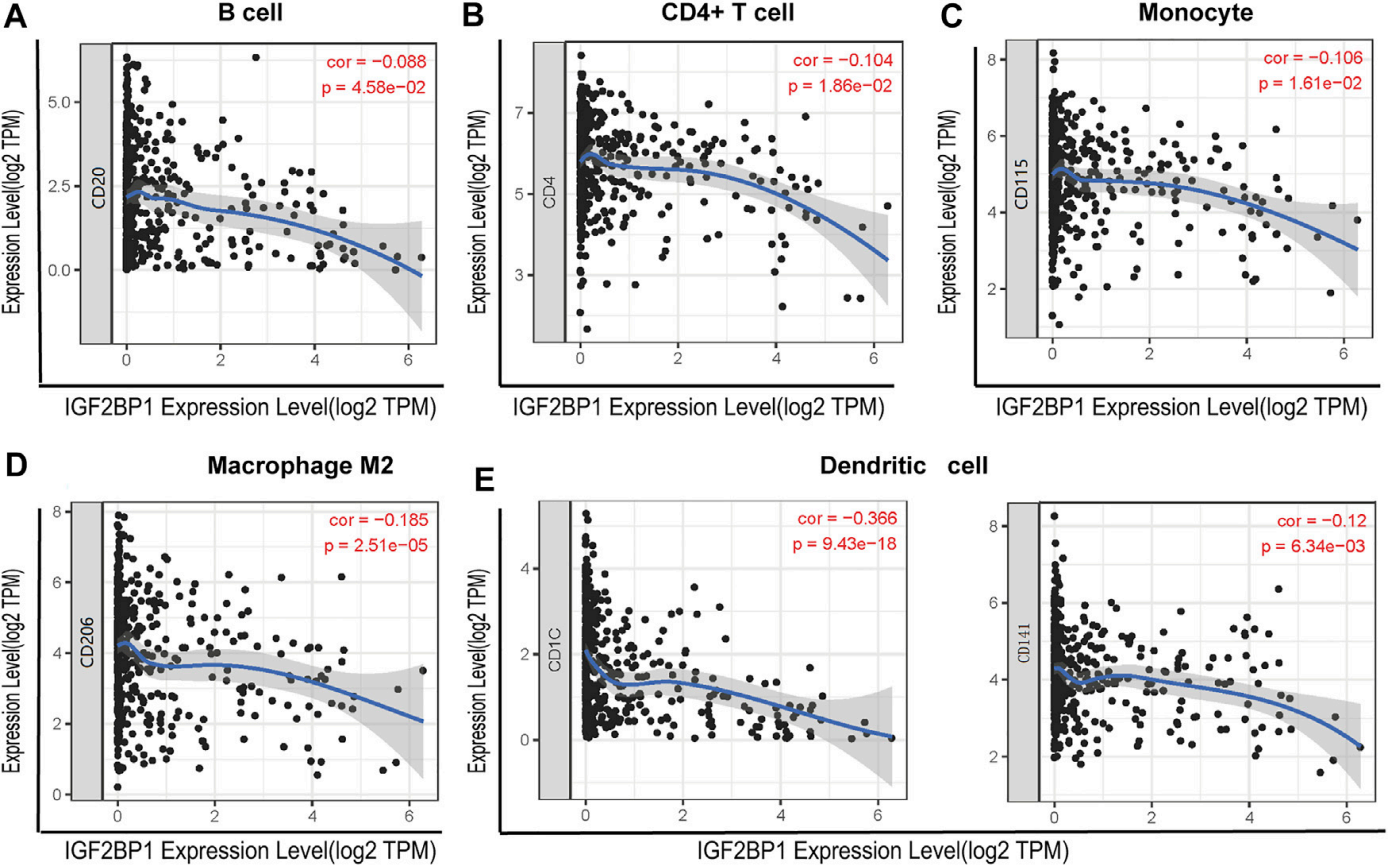
Correlation between *IGF2BP1* expression and the expression of marker genes of infiltrating immune cells in LUAD using the TIMER database. **(A)** B cell (CD20); **(B)** CD4^+^ T cell (CD4); **(C)** monocytes (CD115); **(D)** macrophage M2 (CD206); and **(E)** dendritic cell (CD1C and CD141). *IGF2BP1* was represented on the *x*-axis with gene symbols, and related marker genes are represented on the *y*-axis as gene symbols. The gene expression level was displayed with log2 RSEM.

To investigate the relationship between *IGF2BP1* and the diverse immune-infiltrating cells, we focused on the correlations between *IGF2BP1* and immune markers of various immune cells of LUAD in the TIMER. The results revealed that the *IGF2BP1* expression level was significantly correlated with immune markers of CD4^+^ T cells (CD4), B cells (CD20), monocytes (CD115), M2 macrophages (CD206), and DCs (CD1C and CD141) in LUAD (Figure 7). We further analyzed the correlation between *IGF2BP1* expression and the marker of B cell (CD20) in LUAD tissues by IHC. The result showed that *IGF2BP1* expression was negatively correlated with CD20 expression in LUAD (Supplementary Figure S3), which was similar to that in TIMER. Together, these results suggested that *IGF2BP1* played an important role in immune cell infiltration in LUAD.

### Correlation of the *IGF2BP1* Gene With Immune Checkpoints, TMB, and MSI

**FIGURE 8:**
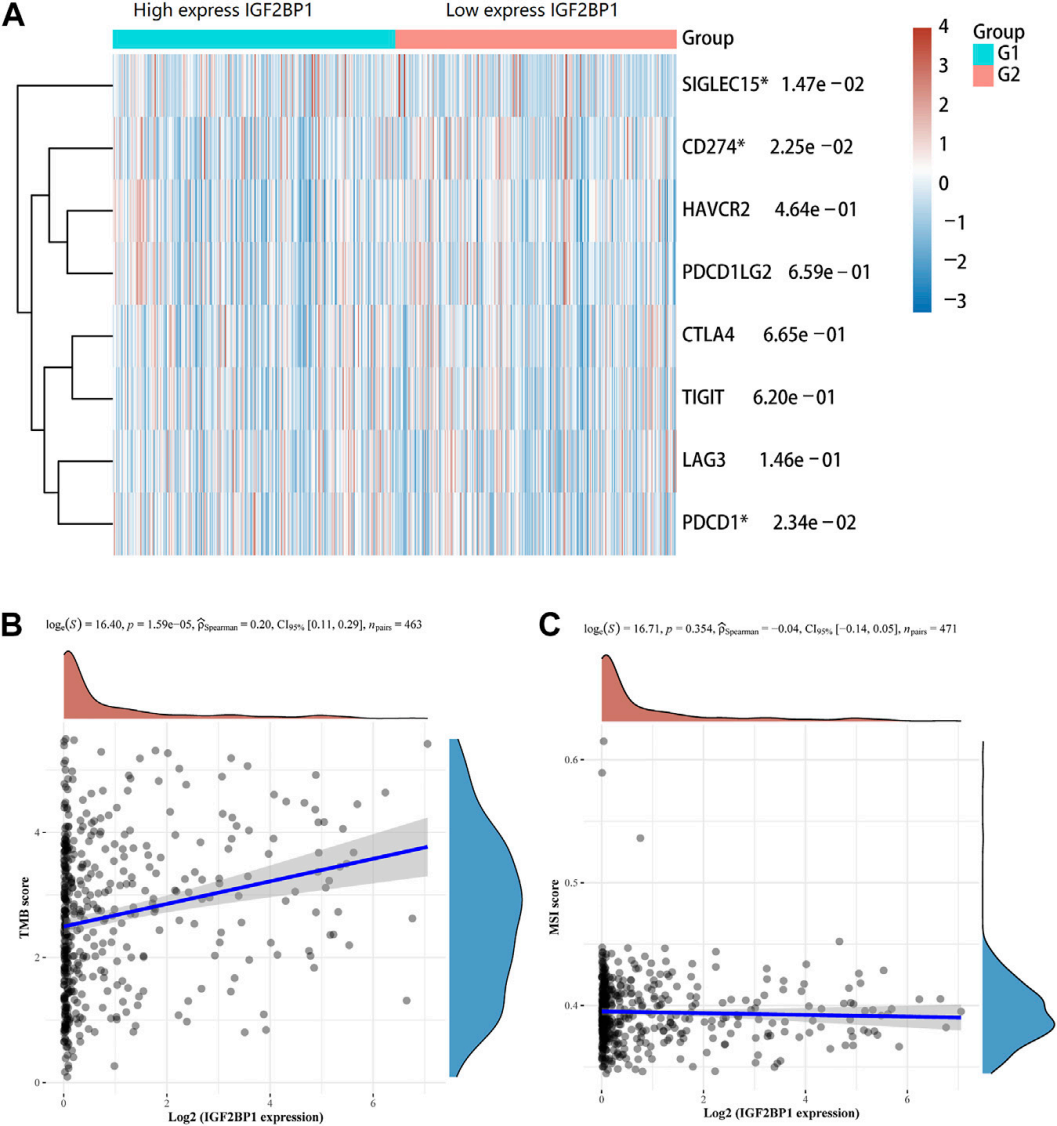
Association of the *IGF2BP1* gene with immune checkpoints, TMB, and MSI in LUAD. **(A)** Correlation of *IGF2BP1* expression with immune checkpoint genes. **(B)** Correlation of *IGF2BP1* expression with tumor mutational burden (TMB). **(C)** Correlation of *IGF2BP1* expression with microsatellite instability (MSI).

Immune checkpoints are the essential regulatory molecules for maintaining self-tolerance, preventing autoimmune response, and minimizing tissue damage by controlling the duration and intensity of the immune responses, which produces effective antitumor immune responses. Our result showed that *IGF2BP1* was correlated with three immune checkpoint genes (*SIGLEC15*, *CD274*, and *PDCD1*) in LUAD (Figure 8A). TMB is defined as the total number of somatic mutations per megabase (Mb) in coding regions of an exon, which is a predictive biomarker for the efficacy of tumor immunotherapy. Our result showed that *IGF2BP1* expression was positively related to TMB (*p* < 0.001) (Figure 8B). Microsatellite (MS) refers to a tandem repeat sequence (1-6 nucleotides) usually located in the intergenic region, promoter, UTR, and coding region. The changes of the MS-DNA structure (mismatches, insertions, and/or deletions) under certain pathological factors could lead to MSI, which is associated with malignant transformation of human cells. But, our result showed that there was no significant correlation between the expression of *IGF2BP1* and MSI (Figure 8C). Our study suggested that *IGF2BP1* might be related to immunotherapy response in LUAD, which could serve as a novel biomarker for predicting the immunotherapy response rate.

### GSEA Analysis and Functional Enrichment of the *IGF2BP1* Gene

**FIGURE 9:**
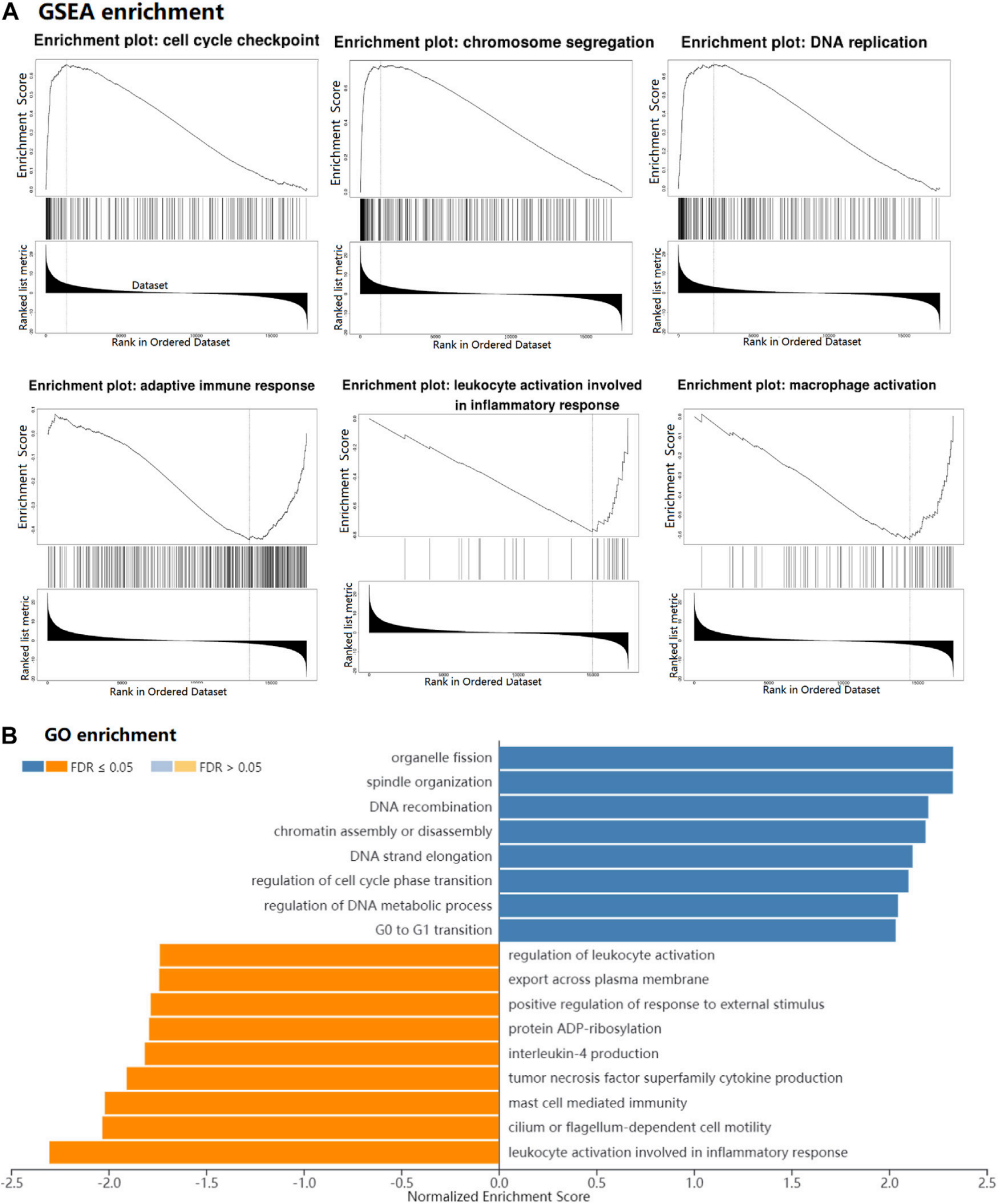
GSEA and functional enrichment of the *IGF2BP1* gene in LUAD. **(A)** GSEA of *IGF2BP1* in LUAD by LinkedOmics. **(B)** GO enrichment of *IGF2BP1* in LUAD. Blue bar means positively associated biological process, and yellow bar means negatively associated biological process.

In our study, GSEA analysis was used to analyze pathway enrichment for the *IGF2BP1* gene. The result showed that *IGF2BP1* was significantly related to the activation of the cell cycle-related pathway (including cell cycle checkpoint, chromosome segregation, and DNA replication) and the inhibition of the immune-related pathway (including adaptive immune response, leukocyte activation, and macrophage activation) in LUAD (Figure 9A). Meanwhile, GO enrichment analysis showed that the cell cycle had a positive correlation with the *IGF2BP1* expression, and immune regulation had a negative correlation with the *IGF2BP1* expression (Figure 9B), which also supported the biological functions of the *IGF2BP1* gene.

## Discussion

m^6^A modification plays an important role in the tumorigenesis and metastasis of various cancers by regulating RNA stability, microRNA processing, mRNA shearing, and translation. Studies have found that m^6^A modification regulators are significantly abnormally expressed in various cancers, which lead to malignant proliferation, migration, invasion, metastasis, and drug resistance (Yang et al., 2019). Recent studies show that the levels of m^6^A-related genes are also associated with the prognosis of lung cancer patients. For instance, Zhuang et al. found that HNRNPC played a critical role in LUAD progression (Zhuang et al., 2020). Zhang et al. also found that HNRNPA2B1 and HNRNPC were closely related to the overall survival of LUAD patients (Wang et al., 2021a). In our study, we aimed to systematically explore the biological function of m^6^A methylation regulators and the relationships with tumor immune in LUAD, which could provide a theoretical basis for making clinical treatment strategies.

We totally selected 20 m^6^A modification regulators, including two “erasers” (*ALKBH5* and *FTO*), eleven “readers” (*HNRNPA2B1*, *HNRNPC*, *IGF2BP1*, *IGF2BP2*, *IGF2BP3*, *RBMX*, *YTHDC1*, *YTHDC2*, *YTHDF1*, *YTHDF2*, and *YTHDF3*), and seven “writers” (*METL14*, *METL3*, *RBM15*, *RBMWTAP*, *VIRMA*, *WTAP*, and *ZC3H13*) for analysis. Among 20 m^6^A modification regulators, 15 m^6^A modification regulators (*ALKBH5*, *FTO*, *HNRNPA2B1*, *HNRNPC*, *IGF2BP1*, *IGF2BP3*, *RBMX*, *YTHDF1*, *YTHDF2*, *METL14*, *METL3*, *RBM15*, *VIRMA*, *WTAP*, and *ZC3H13*) were significantly abnormally expressed in LUAD tissues. Previous studies have shown that m^6^A modification regulators, including m^6^A writers [METTL3 (Lin et al., 2016) and METTL4 (Gong et al., 2020; Liu et al., 2020; Wang et al., 2021b; Nombela et al., 2021)], m^6^A erasers [FTO (Liu et al., 2018; Chen and Du, 2019; Tang et al., 2020; Nombela et al., 2021) and ALKBH5 (Jin et al., 2020)], and m^6^A readers [YTHs (Jin et al., 2020) and IGF2BPs (Degrauwe et al., 2016; Müller et al., 2019)], play important roles in the occurrence and development of various cancers.

Then, Kaplan–Meier survival analysis showed the expressions of *IGF2BP1*, *IGF2BP3*, *HNRNPC*, and *HNRNPA2B1* were significantly associated with the poor prognosis of LUAD. Furthermore, multivariate Cox regression analysis revealed that only *IGF2BP1* remained independently associated with the prognosis of LUAD after adjusting the clinical variables (gender, age, pT/pN stage, and smoking history). The nomogram analysis also showed that *IGF2BP1* had a predictive value for overall survival (1, 3, and 5 years) in LUAD patients. The result suggested that *IGF2BP1* was an independent risk factor of poor prognosis in LUAD. IGF2BPs family proteins (including IGF2BP1, IGF2BP2, and IGF2BP3) are a novel discovered m^6^A-binding proteins. Studies have shown that the IGF2BPs expressions are abnormally expressed in a variety of cancers, which regulate tumor progression by a variety of molecular mechanisms (Degrauwe et al., 2016). The increased expression of IGF2BP1 is significantly related to the poor prognosis of ovarian cancer, liver cancer, and lung cancer (Huang et al., 2019; Müller et al., 2019; Zhang et al., 2020). As an RNA-binding protein, IGF2BP1 can also affect the function of the target RNA by binding to the RNA. Recent studies have found that IGF2BP1 can bind to lncRNA LIN28B and activate its function to promote the proliferation and metastasis in LUAD cells (Wang et al., 2019). Currently, there are still few studies on IGF2BP1 in LUAD, and a synthetical study of IGF2BP1 in LUAD is needed to perform.

We further explored the mutation rate of m^6^A modification regulators in 1,989 LUAD samples using the cBioPortal database. Consistent with the previous studies, our result showed that the mutation rates of the m^6^A modification regulators in LUAD were not high (<10%), which suggested that m^6^A modification regulators might not only influence tumorigenesis of LUAD through gene mutation.

Tumor-infiltrating immune cells are closely associated with the clinical outcome of cancers. Therefore, the association between *IGF2BP1* and LUAD immune infiltration were further explored. The result showed that *IGF2BP1* expression was related to immune infiltration of macrophage M0/1/2, T cell CD4^+^ memory resting/activation, mast cell activation, monocyte, myeloid dendritic cell resting, T cell follicular helper, and B cell memory. Moreover, we found that the *IGF2BP1* expression level was significantly correlated with immune markers of CD4^+^ T cells (CD4), B cells (CD20), monocytes (CD115), M2 macrophages (CD206), and DCs (CD1C and CD141) in LUAD. Macrophages, including macrophage M0 and macrophage M2, were shown to be the most abundant immune cells in non-small cell lung cancer (House et al., 2020). Recent studies have shown that the degree of immune cell infiltration is significantly related to the prognosis of non-small cell lung cancer. Patients with low immune cell infiltration have lower cytotoxic activity and lower expression levels of MHC-I and immune checkpoints, which may lead to the possibility of immune escape. Meanwhile, patients with a high degree of immune cell infiltration may have a better immune response (Mi et al., 2020). Taken together, our findings indicate that IGF2BP1 plays an important role in regulating tumor-infiltration of immune cells in LUAD.

Immunotherapy is a hot spot of lung cancer research studies. The roles of m^6^A in tumor immunity and cell cycle regulation have been highly interested by researchers. The m^6^A modification regulators (such as FTO) play important roles in the PD-1/PD-L1 inhibitor tumor immunotherapy (Yang et al., 2019; Li et al., 2020). Immune checkpoints are the essential regulatory molecules to control the duration and intensity of immune responses. In our study, *IGF2BP1* expression was significantly associated with three immune checkpoint genes (*SIGLEC15*, *CD274*, and *PDCD1*). TMB can be used as a predictive biomarker for the efficacy of immune checkpoint inhibitor therapy. We found that *IGF2BP1* expression was significantly correlated with the TMB of LUAD. So far, there are few studies on *IGF2BP1* and immune checkpoint and TMB. Our study was the first to report the correlation of *IGF2BP1* with immune checkpoints and TMB in LUAD, which might be important for immunotherapy of LUAD.

Furthermore, the GSEA showed that *IGF2BP1* was significantly related to the inhibition of adaptive immune response, leukocyte activation, and macrophage activations in LUAD. At present, there are lack of studies regarding the correlation between *IGF2BP1* and LUAD. Further study is necessary to explore the role of IGF2BP1 involvement in the immune response in LUAD.

There are some limitations in our study. First, the study was a retrospective bioinformatic analysis, which could have certain general bias. Second, other studies are necessary to distinguish the effects of *IGF2BP1* on the tumor immune infiltration pathway. Third, further studies are necessary to verify the role played by IGF2BP1 in LUAD.

In conclusion, our work systemically elucidated the role of m^6^A modification regulators in LUAD. Among them, *IGF2BP1* was independently related to the prognosis of LUAD. Moreover, *IGF2BP1* expression was significantly related to immune infiltration, TMB, and the expressions of immune checkpoints. These findings suggest that *IGF2BP1* may be a potential independent biomarker for LUAD prognosis and the status of tumor immunity.

## Data Availability

The original contributions presented in the study are included in the article/Supplementary Material, further inquiries can be directed to the corresponding authors.

## Supplementary Material

Supplementary Figure 1 Protein level of IGF2BP1 in LUAD and normal tissues by IHC.

Supplementary Figure 2 Kaplan–Meier survival curves comparing the high and low expression of m^6^A modification regulators in LUAD using PrognoScan, based on the GEO database (GSE31210) (*n* = 204).

Supplementary Figure 3 Protein level of IGF2BP1 and CD20 in LUAD and normal tissues by IHC (100 ×  magnification).
