# Supplementary figures and images for: RNA-Binding Protein IGF2BP1 Associated With Prognosis and Immunotherapy Response in Lung Adenocarcinoma
Source: Front Genet. 2022 Jan 27;13:777399. doi: 10.3389/fgene.2022.777399 (PMC8830935; doi:10.3389/fgene.2022.777399)

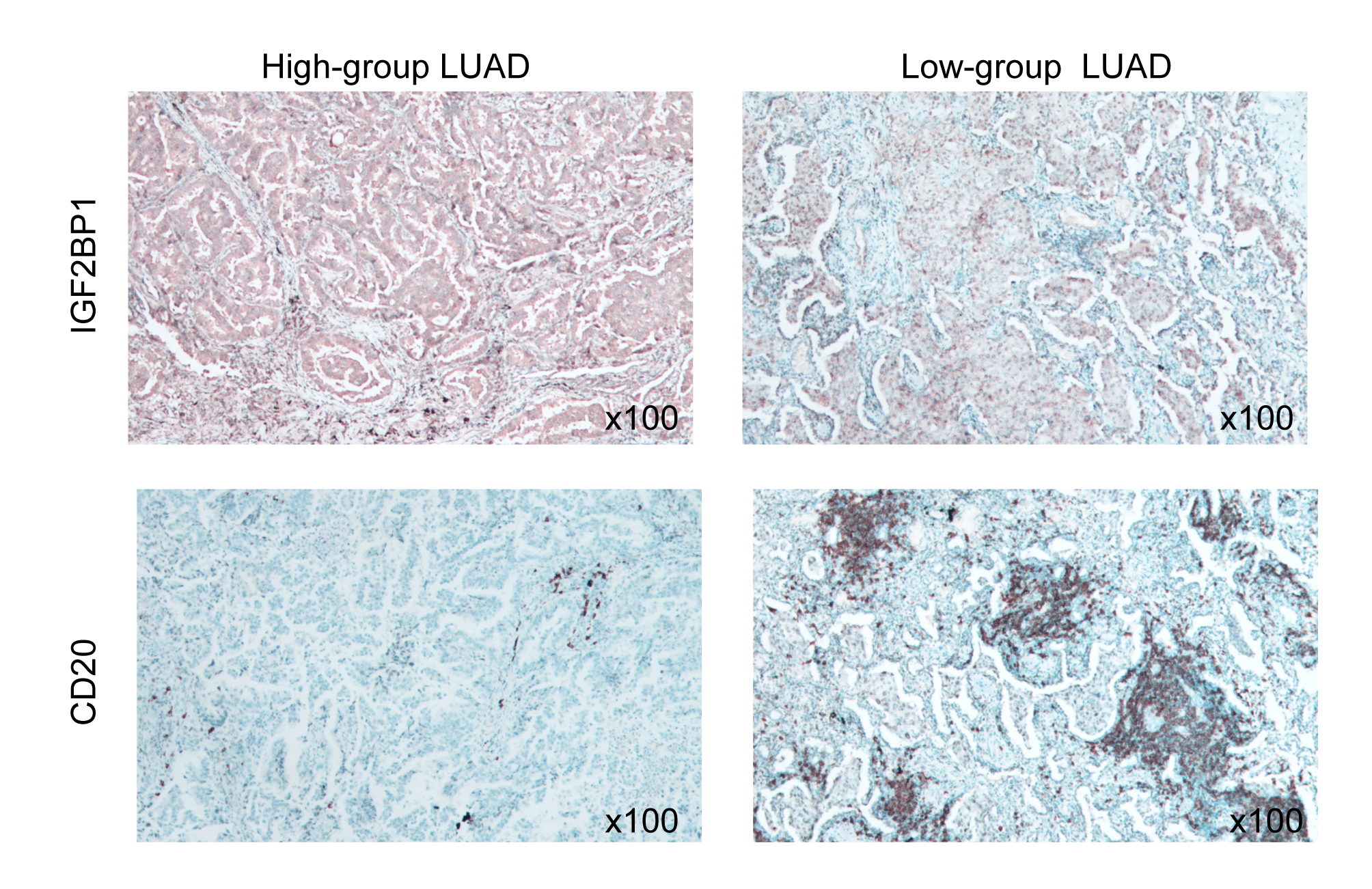

Supplement: Supplementary file 1 [file Image3.TIF]

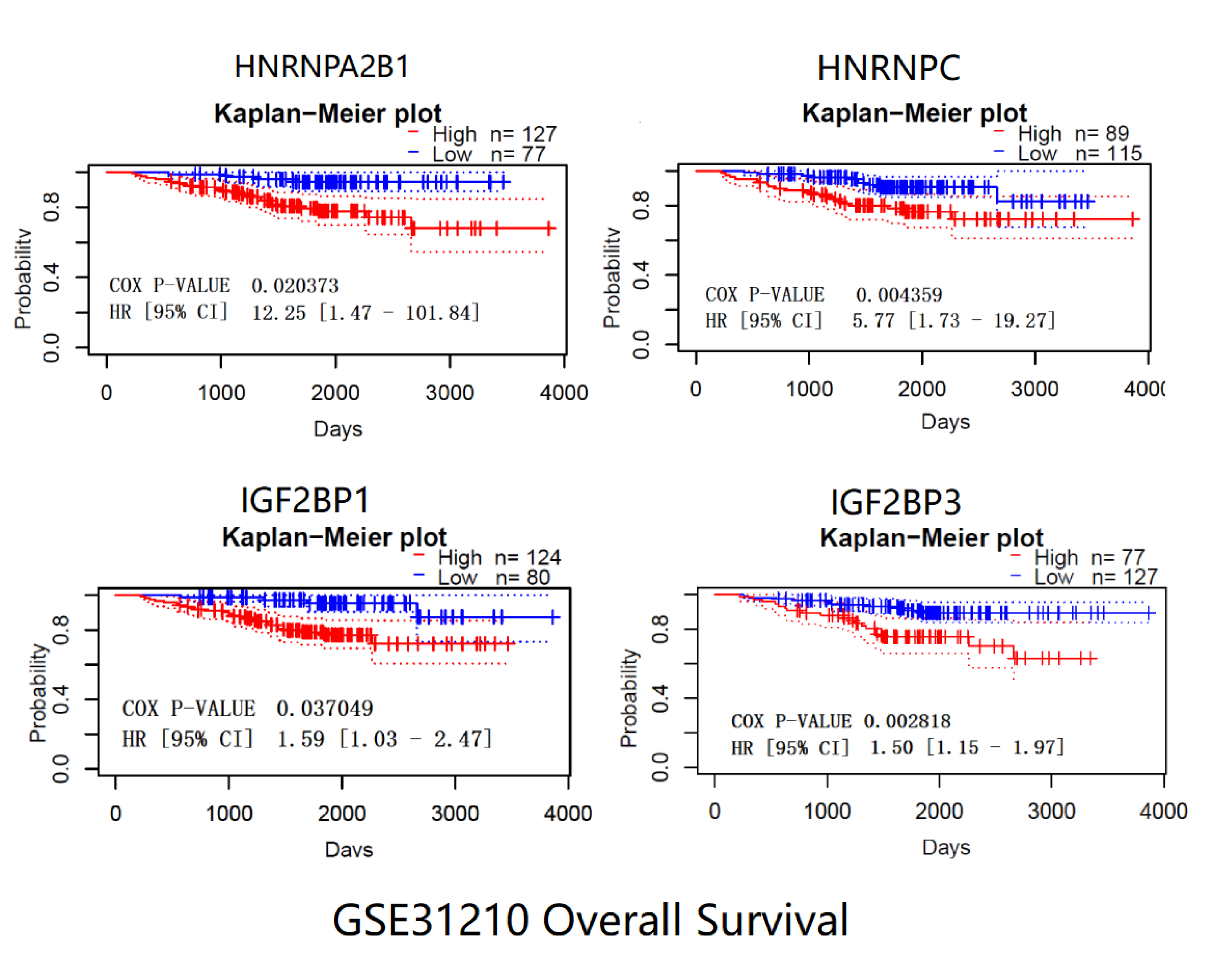

Supplement: Supplementary file 2 [file Image2.TIF]

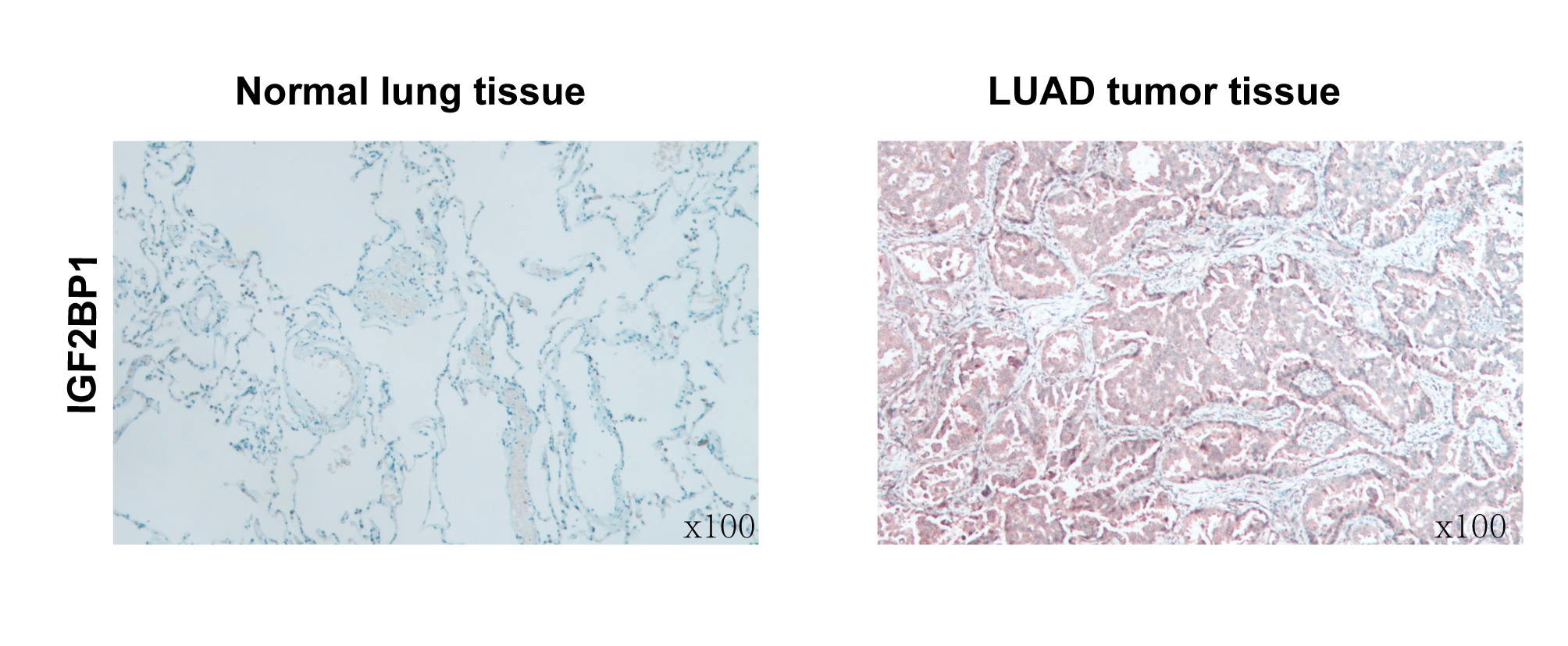

Supplement: Supplementary file 3 [file Image1.TIF]
